# 68477 Pancreatic cancer cell extracellular vesicles drive the unfolded protein response in recipient normal pancreatic cells.

**DOI:** 10.1017/cts.2021.445

**Published:** 2021-03-30

**Authors:** Charles Hinzman, Shivani Bansal, Yaoxiang Li, Jose Trevino, Partha Banerjee, Amrita Cheema

**Affiliations:** 1 Georgetown University Medical Center; 2 University of Florida College of Medicine

## Abstract

ABSTRACT IMPACT: This study advances our understanding of potentially key drivers in the early formation of pancreatic cancer, a disease with few treatment options and poor patient outcomes. OBJECTIVES/GOALS: Patients diagnosed with pancreatic ductal adenocarcinoma (PDAC) have a 5-year survival rate of ˜9%. A key driver of poor patient outcomes is late-stage diagnosis. A better understanding of PDAC onset is needed. This study was developed to understand how extracellular vesicles may be involved in the early formation of PDAC. METHODS/STUDY POPULATION: Extracellular vesicles (EVs) were isolated from several human PDAC and normal pancreatic cell lines, using ultracentrifugation with filtration or size exclusion chromatography. We next treated normal pancreatic cell lines with cancer cell EVs (cEVs). Next generation sequencing was used to measure global gene expression changes after treatment. Validations were performed using qPCR and luciferase activity assays. Multi-omics characterization of EVs was accomplished using mass spectrometry based proteomics, metabolomics and lipidomics analysis. RESULTS/ANTICIPATED RESULTS: We found that normal cells upregulated a variety of stress response pathways in response to cEVs. Lipid synthesis was also severely downregulated in these cells. We further validated activation of the unfolded protein response (UPR) in normal cells treated with cEVs. Multi-omics characterization of cEVs identified several enriched proteins, lipids and metabolites which may play a role in the activation of the UPR. DISCUSSION/SIGNIFICANCE OF FINDINGS: Our results indicate that cEVs induce stress, and in particular the UPR, in normal pancreatic cells. Long-term UPR can impact a variety of cancer hallmarks. The UPR can mediate progression of pancreatic intraepithelial neoplasia (PanIN) to PDAC. Our results highlight a potential role for cEVs to alter the function of normal cells, aiding disease onset.

